# The MHC Class II transactivator CIITA inhibits the persistent activation of NF-kB by Tax-1

**DOI:** 10.1186/1742-4690-12-S1-P58

**Published:** 2015-08-28

**Authors:** Greta Forlani, Rawan Abdallah, Luisa Guidali, Roberto S Accolla, Giovanna Tosi

**Affiliations:** 1 Department of Surgical and Morphological Sciences, University of Insubria, Varese, Italy; 2 Department of The oretical and Applied Sciences, University of Insubria, Varese, Italy

## 

Human T-cell Lymphotropic Virus type-1 (HTLV-1) is the causative agent of an aggressive malignancy of CD4+ T lymphocytes. Many studies have shown that the constitutive activation of NF-kB pathway by the viral transactivator Tax-1 is crucial for T-cell transformation. We previously identified the cellular factor Class II transactivator (CIITA), the master regulator of Major Histo compatibility Complex Class II gene transcription, as a restriction factor inhibiting HTLV-1 replication by blocking Tax-1-mediated activation of the viral LTR promoter (1). Here we show that CIITA suppresses also the activation of the canonical NF-kB pathway by Tax-1 and mapped the region of CIITA mediating this effect. CIITA affects the subcellular localization of Tax-1, which is mostly retained in the cytoplasm and this correlates with an impaired migration of Rel Ain to the nucleus. By using nuclear and cytoplasmic deletion mutants of CIITA, we demonstrate that CIITA suppresses the activation of NF-kB by Tax-1 in both the subcellular compartments. Interestingly, CIITA binds to Tax-1 in vivo without preventing the binding of Tax-1 to both IKKg and RelA. Nevertheless, Tax-1-induced IKK kinase activity is affected in the presence of CIITA as demonstrated by impaired phosphorylation of IkB inhibitor, which is responsible for the impaired migration of RelA into the nucleus. Thus, the inactive p50/RelA heterodimer is trapped in the cytoplasm and this results in the suppression of the activation of NF-kB responsive genes by Tax-1. Overall, these findings indicate that CIITA has evolved as a versatile molecule that, besides inhibiting viral gene expression promoted by Tax-1, it might counteract also Tax-1 transforming activity. Thus, unveiling the molecular basis of CIITA-mediated Tax-1 inhibition may be important in defining new strategies to control HTLV-1 spreading and on cogenic potential.

